# CD73 is expressed by inflammatory Th17 cells in experimental autoimmune encephalomyelitis but does not limit differentiation or pathogenesis

**DOI:** 10.1371/journal.pone.0173655

**Published:** 2017-03-13

**Authors:** Gerard Hernandez-Mir, Mandy J. McGeachy

**Affiliations:** Division of Rheumatology & Clinical Immunology, Department of Medicine, University of Pittsburgh, Pittsburgh, Pennsylvania, United States of America; University of Texas at San Antonio, UNITED STATES

## Abstract

CD73 works together with CD39 to convert extracellular ATP to immunoregulatory adenosine, thus inhibiting inflammation. TGFβ-mediated CD73 expression on ‘regulatory’ Th17 cells limits their ability to eradicate tumors, similar to the immunosuppressive mechanism described for CD73 on Tregs. However, CD73 is also expressed on Th17 cells thought to be inflammatory in Crohn’s disease. CD73 has previously been reported to contribute to inflammation in the central nervous system (CNS). In experimental autoimmune encephalomyelitis (EAE), we found that inflammatory cytokine-producing Th17 cells showed increased CD73 expression as disease progressed. We therefore hypothesized that CD73 could be important for limiting the expansion or pathogenic function of Th17 cells in autoimmune inflammation of the CNS. Surprisingly, EAE development was not enhanced or inhibited by CD73 deficiency; there was correspondingly no difference in induction of Th17-associated cytokines IL-17, IFNγ or GM-CSF or recruitment of either inflammatory or regulatory cells to the central nervous system. We confirmed that CD73 was similarly not required for differentiation of Th17 cells *in vitro*. These data show that while CD73 expression is regulated during EAE, this enzyme is not absolutely required to either promote or limit Th17 cell expansion or EAE severity.

## Introduction

Th17 cells produce cytokines including IL-17, GM-CSF and IFNγ that orchestrate immune and tissue inflammatory responses resulting in recruitment and activation of myeloid cells, as well as production of antimicrobial peptides and matrix metalloproteinases. These responses are beneficial in controlling extracellular bacteria and fungal pathogens such as *Staphylococcus aureus* and *Candida albicans*, as well as promoting wound healing following resolution of the infection[1, 2]. However, when Th17 responses are dysregulated or are inappropriately induced against commensal microbes or self-proteins, this can result in chronic inflammatory diseases exemplified by psoriasis, inflammatory bowel disease and multiple sclerosis[3]. Hence there needs to be a critical balance between promoting Th17-mediated inflammation when it is beneficial, while regulating Th17 cells to prevent immunopathology.

Perhaps because of the propensity of Th17 cells to evoke potent inflammation, multiple mechanisms exist to regulate the expansion or function of Th17 cells. During differentiation, exposure to cytokines including IL-2, IL-27 and IFNγ inhibit development of Th17 cells[4]. TGFβ is required for early Th17 cell activation, and T cells deficient in TGFβ or its receptor show impaired Th17 development and function *in vivo*[5, 6]. However, Th17 cells that receive sustained stimulation from TGFβ are non-pathogenic in function, despite high production of IL-17[7, 8]. These non-pathogenic Th17 cells have also been termed ‘regulatory’ Th17 cells due to their capacity to suppress inflammation through production of IL-10, although there are multiple mechanisms determining their non-pathogenic phenotype[9, 10].

One of the molecules expressed both by ‘regulatory’ Th17 cells and Foxp3^+^ regulatory T cells is the enzyme Ecto-5’-nucleotidase, also called CD73[11–13]. TGFβ along with STAT3 activation induces expression of CD73 along with Ectonucleoside triphosphate diphosphohydrolase-1, also known as CD39[13]. These two enzymes work together: CD39 converts extracellular ATP to AMP intermediates, which are then converted to adenosine by CD73. Generation of adenosine by CD73 on Tregs has been shown to have immunosuppressive functions on Th1 cells[13]. ‘Regulatory’ Th17 cells expressing high CD73 also impaired anti-tumor responses in mouse models[13, 14]. Corresponding with immunosuppression, high CD73 expression is associated with poor prognosis in human cancers[15].

The roles of CD73 in regulating autoimmune inflammation are less clear. Inflammatory Th17 cells in the intestine of Crohn’s disease patients were found to express CD73[16]; whether this had any functional consequence or association with disease severity was not determined. CD73 expression has been reported in multiple sclerosis (MS) brain lesions[17]. Experimental autoimmune encephalomyelitis (EAE) is a widely used model of CNS-targeted inflammation mediated by Th17 cells. As predicted by the immunosuppressive roles of adenosine, mice deficient in adenosine receptor A2A show accelerated onset and severity of EAE, along with increased production of inflammatory cytokines[18, 19]. Hence the prediction would be that CD73^-/-^ mice, having reduced ability to generate adenosine, might develop EAE with increased severity. However, contradictory findings have been reported in regards to CD73 and adenosine in the EAE model. In one report, CD73^-/-^ mice were protected from active EAE, while transferred CD73^-/-^ T cells induced more severe disease in wildtype (WT) recipients[20]. In contrast to the immunosuppressive effects of adenosine during EAE induction, adenosine signaling during later phases of EAE is thought to promote immune cell infiltration of the CNS through upregulation of chemokines[21]. Despite evidence for CD73 expression by Th17 cells, the role of CD73 in Th17 differentiation has not been carefully analyzed. We therefore set out to first confirm and then extend previous data by investigating expression of CD73 and its role during differentiation of Th17 cells.

## Results

### CD73 expression on different T cell subsets

**Fig 1:**
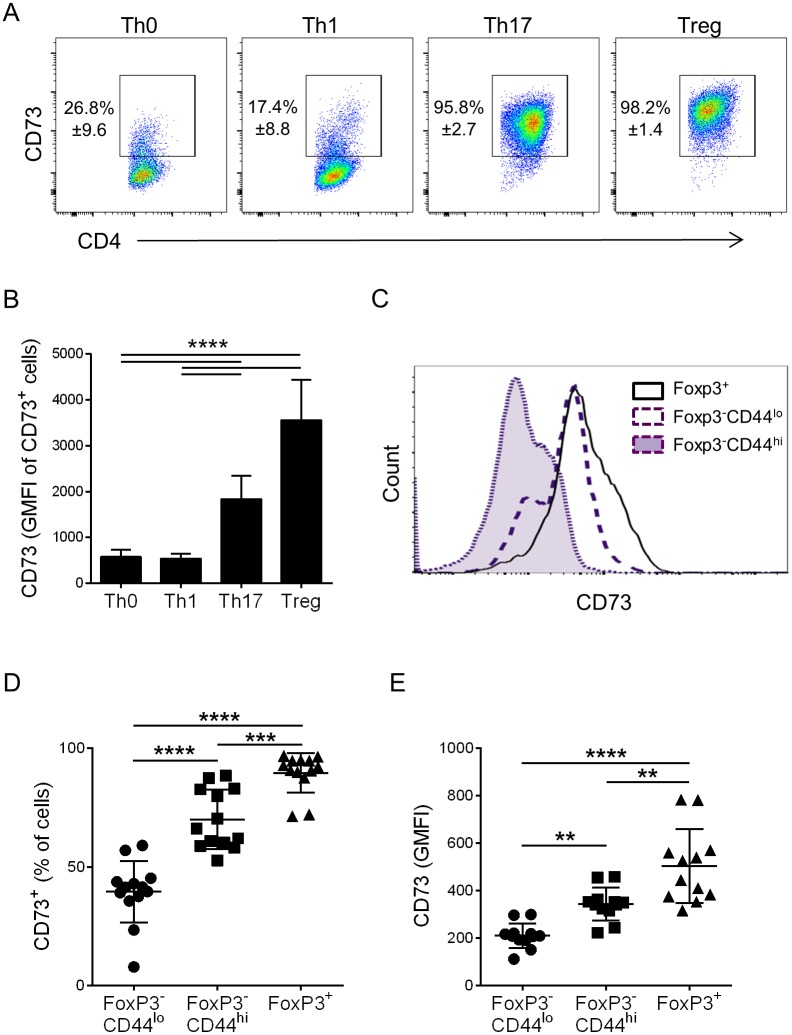
CD73 expression on T helper cells *in vitro* and *in vivo*. A: CD73 expression on indicated live CD4^+^ T helper cell subsets after three days of ulture *in vitro*, numbers indicate mean % +/- S.D. from three separate experiments. B: Mean level of CD73 expression measured as Geometric Mean Fluorescence Intensity of gated live CD4^+^CD73^+^ cells, from three experiments. C: Representative histogram of CD73 expression by Tregs (FoxP3^+^), naïve T cells (FoxP3^-^ CD44^lo^), or effector/memory (FoxP3^-^ CD44^hi^) T cells in LN from naïve C57BL/6 mice. D: Mean percentage of CD73^+^ cells and E: CD73 Geometric Mean Fluorescence Intensity of populations indicated in C; data pooled from 4 independent experiments (each point represents a separate mouse). Error bars indicate S.D.

CD73 expression is increased on TGFβ-stimulated Th17 cells and regulatory T cells *in vitro*, while remaining low on Th0 and Th1 cells (Fig 1A), confirming previous reports. *In vitro*-generated regulatory T cells expressed higher levels of CD73 than Th17 cells (Fig 1B). *In vivo*, Foxp3^+^ Tregs in naïve mice were also CD73^+^, and showed higher expression of CD73 than Foxp3^-^CD44^hi^ effector/memory cells from the same animals (Fig 1C–1E). Interestingly, Foxp3^-^CD44^lo^ cells, which are typically considered naïve cells, also showed some expression of CD73, albeit lower percentages and levels of expression per cell (Fig 1C–1E).

### Th17 cells upregulate expression of CD73 during EAE progression

**Fig 2:**
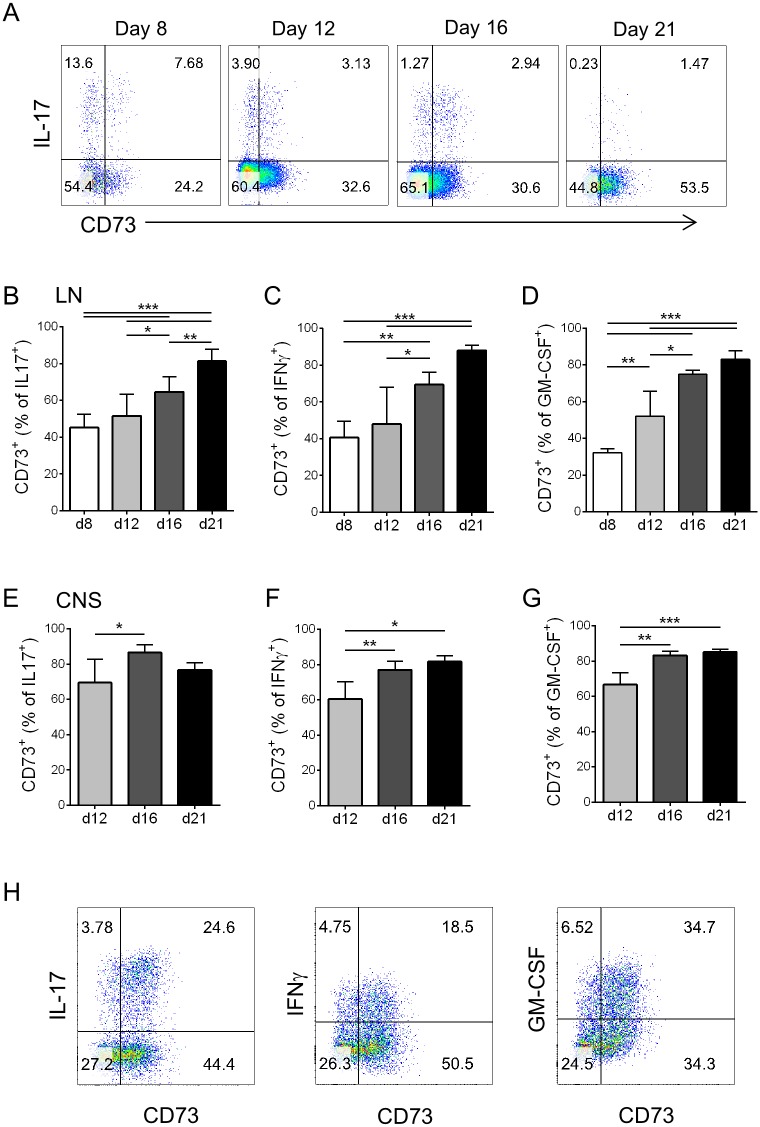
Th17 cells increase expression of CD73 during EAE progression. A-D: Analysis of co-expression of CD73 on cytokine-producing cells in draining LN, gated on live CD4^+^ cells, at indicated time-points post EAE induction. A: Representative FACS plots. B: Percentage of IL-17^+^ T cells that express CD73; C: Percentage of IFNγ^+^ T cells that express CD73; D: Percentage of GM-CSF^+^ T cells that express CD73. E-H: Analysis of co-expression of CD73 on cytokine-producing cells in CNS, gated on live CD4^+^ cells, at indicated time-points post EAE induction. E: Percentage of IL-17^+^ T cells that express CD73; F: Percentage of IFNγ^+^ T cells that express CD73; G: Percentage of GM-CSF^+^ T cells that express CD73. H: Representative FACS plots showing CD73 and cytokine staining in live CD4^+^ T cells from CNS on day 16 of EAE. Values in graphs correspond to mean +/- S.D. n = 5–13 mice/time-point pooled from 2–3 experiments (except day 16 GM-CSF is from one experiment).

These data suggest that CD73 is not only a marker of regulatory cells but is also expressed on a large proportion of effector/memory cells. Hence we investigated expression of CD73 on Th17 cells during induction and effector phases of the inflammatory autoimmune response in EAE. At the peak of T cell activation in the draining LN (day 8 following immunization), CD73 was expressed on almost half of IL-17^+^ cells (Fig 2A and 2B). Interestingly, the proportion of IL-17^+^ cells that expressed CD73 increased as the response progressed (Fig 2A and 2B). The expression of CD73 on LN IFNγ^+^ cells showed a similar increase as the EAE response progressed (Fig 2C), and GM-CSF^+^ cells followed the same pattern (Fig 2D). Corresponding with the increasing expression of CD73 by cytokine-producing cells in LN, the effector cells found in the CNS also contained high proportions of cytokine-producing cells that co-expressed CD73 (Fig 2E–2H), which increased from onset through peak and chronic phases of disease.

### CD73 does not influence Th17 differentiation *in vitro*

**Fig 3:**
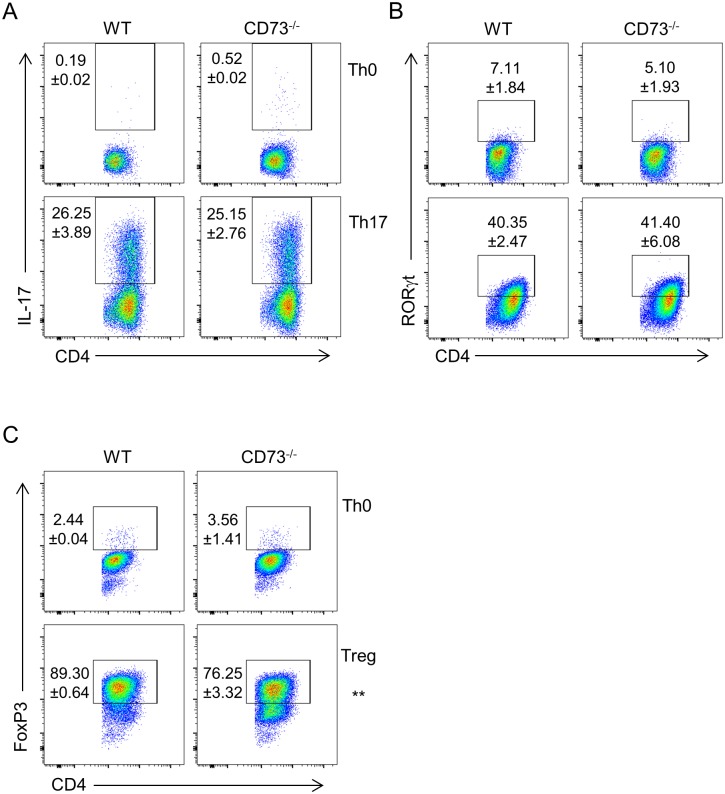
CD73 does not influence Th17 differentiation *in vitro*. A, B: CD4^+^ T cells from WT or CD73^-/-^ mice were cultured under Th0 (top panels) or Th17 (lower panels) differentiating conditions for three days, then expression of IL-17 (A) and RORγt (B) were analyzed. C. Expression of FoxP3 in WT and CD73^-/-^ Th0 (top panel) and Tregs (lower panel) at day 3 of differentiation. Numbers indicate mean % +/- S.D. of gated cells (n = 2), representative of three independent experiments with similar results.

Since CD73 is induced on both in vitro-generated ‘regulatory’ Th17 cells and *in vivo* inflammatory Th17 cells, as well as Tregs, we tested whether CD73 plays any role in early differentiation of these cells. WT and CD73^-/-^ T cells were activated with anti-CD3 in presence of Th17-promoting cytokines. Induction of IL-17 and RORγt were comparable in absence of CD73 (Fig 3A and 3B). However, we did observe a small but significant decrease in the percentage of Foxp3^+^ cells when CD73^-/-^ T cells were activated in presence of TGFβ and IL-2 (Fig 3C).

### EAE clinical course is not affected by CD73 deficiency

**Fig 4:**
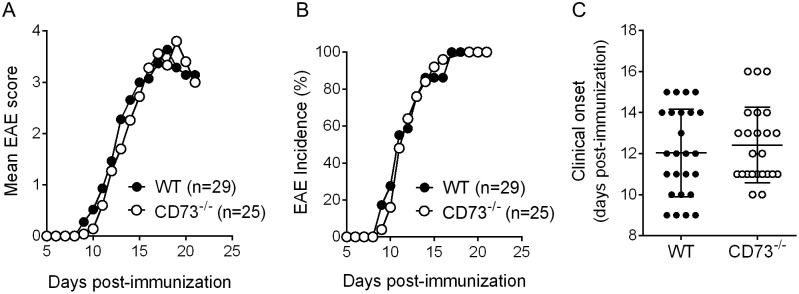
EAE clinical course is not affected by CD73 deficiency. A: Mean clinical scores following EAE induction in WT and CD73^-/-^ mice. B: Percentage of mice that had developed EAE clinical signs on indicated days after EAE induction. C: Day of EAE onset in WT and CD73^-/-^ mice that developed signs of EAE by day 16 post-immunization (mean +/- S.D.). Data pooled from four independent experiments.

The relatively high expression of CD73 on inflammatory T cells during induction and onset of EAE suggests that CD73 may play a role in promoting rather than limiting Th17 function. Conversely, the increased expression of CD73 on these cells as the response progressed and stabilized in terms of clinical disease supports a potential limiting role of this molecule. We therefore tested the requirement for CD73 in autoimmune pathogenic Th17 cell function by immunizing WT and CD73^-/-^ mice with MOG(35–55) to induce EAE. Unexpectedly, there was no difference in severity of EAE in CD73^-/-^ mice (Fig 4A). Similarly, incidence of EAE and day of onset of clinical signs was not different in absence of CD73, although there was a slight non-significant trend towards delayed onset in CD73^-/-^ mice (Fig 4B and 4C).

### Th17 and Treg cell frequency and recruitment to CNS are unaltered by CD73 deficiency

**Fig 5:**
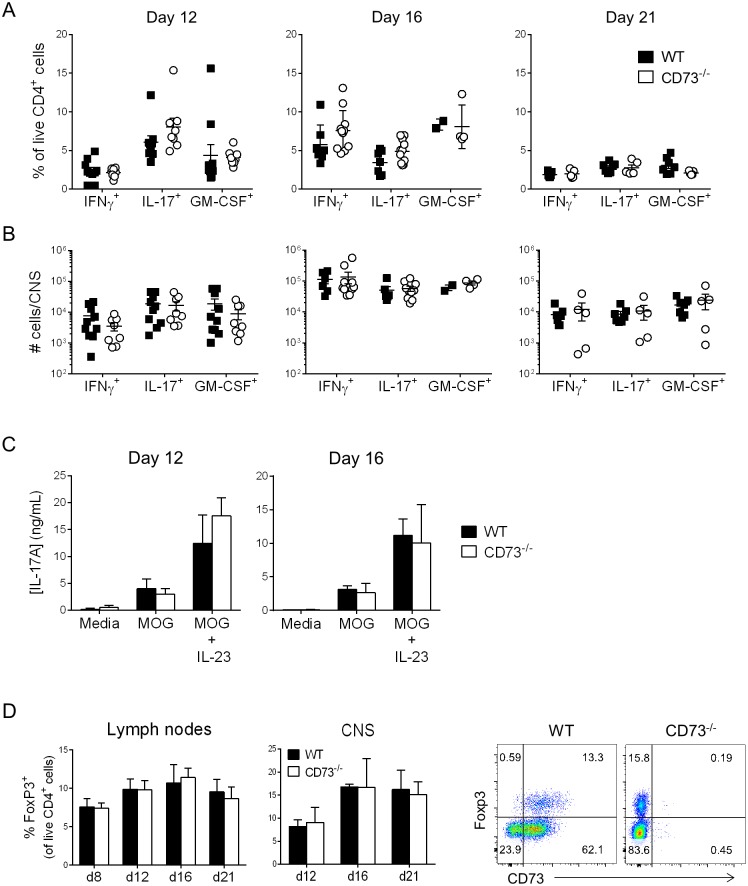
Th17 and Treg cells are similar in WT and CD73^-/-^ during EAE. Cytokine production and Treg frequency were analyzed by FACS in draining LN and CNS of WT and CD73^-/-^ mice at day 12 (onset), day 16 (peak) and day 21 (chronic/resolution) phases of EAE. A: Frequencies of IL-17, IFNγ and GM-CSF expressing T cells, analyzed in draining lymph nodes on indicated days after EAE induction. B: Numbers of IL-17, IFNγ and GM-CSF expressing T cells infiltrating the CNS at indicated time points after immunization. A-B show mean +/- SEM of pooled data, each point represents an individual mouse. C: Cells from draining lymph nodes at days 12 (n = 4-5/group) and 16 (n = 2-3/group) post-immunization were re-challenged *in vitro* with MOG(35–55) for three days in the presence/absence of IL-23 (20ng/mL), and IL-17 expression was measured by ELISA. D: Percentage of Tregs in the draining lymph nodes and CNS at indicated timepoints of EAE, FACS plots show representative staining of Foxp3 and CD73 in CNS on day 16 post-immunization, shown as mean +/- S.D. from 4–7 mice/group except day 16 WT has n = 2 mice. Data are representative of two-three independent experiments with similar results.

It has previously been reported that CD73 expression in the CNS promotes entry of effector cells through induction of chemokines[20, 21], hence it was possible that enhanced activation of Th17 cells was mitigated by reduced entry of these cells into CNS. However, a close examination of cytokine producing T cells revealed no significant differences in frequencies of cells producing IL-17, IFNγ or GM-CSF in LN (Fig 5A) or CNS (Fig 5B) over all phases of the EAE disease course. These data were determined by intracellular cytokine staining following non-specific stimulation with PMA and ionomycin. We also cultured LN cells from WT and CD73^-/-^ mice, taken at onset and peak of EAE, with MOG(35–55) and MOG(35–55) plus IL-23 to promote IL-17 and tested secretion by ELISA. Similar to the flow cytometry results, MOG-induced production of IL-17 was not different (Fig 5C), confirming that priming of the antigen specific Th17 response was not affected by CD73 deficiency. Frequencies of Foxp3^+^ regulatory T cells in LN and CNS were also not affected by CD73 deficiency (Fig 5D). Together, this data corresponds with the clinical scores to confirm that CD73 does not play a critical role in promoting or limiting the inflammatory response induced during EAE.

## Discussion

The data reported here confirmed previous reports that Th17 cells differentiated in the presence of TGFβ express CD73[13]. We also demonstrated that a large proportion of Th17 cells expressed CD73 during EAE induction, and this increased as EAE progressed. More accurately, CD73 expression was similar on IL-17^+^, IFNγ^+^ and GM-CSF^+^ CD4^+^ T cell populations; we group these together as ‘Th17’ since multiple studies show that all three of these cytokines are expressed by Th17 cells in an IL-23 dependent fashion in the EAE model[22–26]. CD73 has previously been described as an immunosuppressive molecule expressed by ‘regulatory’ Th17 cells[13]. It is important to note that while sustained high concentrations of TGFβ induce a non-pathogenic Th17 phenotype, TGFβ and STAT3 are also required for differentiation of inflammatory Th17 cells *in vivo*. Hence, CD73 expression by Th17 cells during EAE corresponds with data showing that TGFβ signals promote Th17 cells to drive EAE induction[5, 6, 27].

The widespread expression of CD73 on Th17 cells in EAE, particularly in the CNS at onset of clinical signs, argued against a purely immunosuppressive role for this molecule in Th17 cell function. However, mice deficient in the receptor for the CD73 product adenosine develop severe EAE with exaggerated cytokine responses[17, 18]. We were therefore surprised to find that CD73^-/-^ mice did not develop exacerbated disease signs following EAE induction. CD73 is also highly expressed on Tregs, which are widely considered to play important roles in controlling autoimmune disease. However, conflicting data exists for the role of Tregs in EAE: on the one hand, depletion of Tregs exacerbated disease severity[28, 29], while on the other hand Tregs were found to be ineffective suppressors of inflammation in the early stages of CNS infiltration[30]. Tregs have also been found to promote Th17 differentiation through absorption of IL-2[31, 32], and *in vitro* can provide a source of TGFβ[5], although this appears to be provided by Th17 cells themselves *in vivo*[6].

Our findings on EAE susceptibility were in contrast to those of Mills et al[20], who found reduced severity of EAE in CD73^-/-^ mice. This was largely attributed to effects of CD73 expressed by CNS-resident cells and on adenosine actions in the CNS to promote expression of chemokines to promote lymphocyte entry to the CNS[20, 21]; T helper cell phenotypes were not intensively analyzed. There are a number of possible reasons for differences in EAE outcome. Different animal facilities have different microbiota communities that can influence outcome of autoimmunity[33]. The EAE induction protocols also vary slightly between labs, and this could result in differences in proportions of Th17 versus Th1 cells induced. In our hands, EAE is associated with strong induction of Th17 cells and the response is dependent on IL-17 and IL-23. We did not find any effect of CD73 deficiency on Th17 induction during any phase of EAE, as measured both by non-specific PMA/ionomycin stimulation and by stimulation with the immunizing antigen MOG(35–55). Differentiation of Th17 cells *in vitro* further supported our unexpected observation that CD73 does not play a dominant role in either inhibiting or promoting Th17 differentiation. Interestingly, Mills et al also reported that mice deficient in the adenosine receptor A2A showed exacerbated EAE with increased IFNγ and proliferation in response to MOG(35–55), supporting the immunosuppressive role of adenosine on Th1 responses[18]. However, IL-17 responses were not impaired in these experiments, corresponding to our current study results and suggesting that the balance between Th17 and Th1 induction in EAE could determine the requirement for CD73 in disease susceptibility.

CD73 works with CD39 to generate adenosine from ATP. Although the focus is often on adenosine as an immunosuppressive molecule, CD39-mediated removal of ATP from the local environment also serves to reduce inflammation[34]: extracellular ATP activates P2X receptors as a damage-associated molecular pattern (DAMP) signal to elicit inflammatory responses such as inflammasome activation and release of IL-1. We did not observe any change in CD39 expression in absence of CD73. Hence, it is likely that the first arm of the CD39/CD73 processing of ATP still acts to control inflammatory responses during EAE. In this context, it was recently reported that Th17 cells have the surprising ability to produce their own IL-1β through activation of the ASC-dependent inflammasome pathway, and ATP is one molecule capable of activating this pathway[35]. Hence, we speculate that Th17 cells may indeed limit their own activation through upregulation of the CD39/CD73 enzyme partners, but that removal of ATP rather than generation of adenosine may play a more important role. Indeed, ‘regulatory’ Th17 cells have been demonstrated to efficiently hydrolyze ATP in a CD39-dependent manner, and CD39 deficiency reduced Th17 cell IL-10 production and increased pathogenic function in colitis[36]. Administration of *Bacteroides fragilis* PSA increases CD39^+^ Tregs and protects from EAE[37]. CD39-deficient mice in this model developed greatly exacerbated disease severity compared to WT controls, and it is possible that this was due to effects on Th17 cells as well as Tregs. Separately, CD39 expressed by dendritic cells during EAE also plays an important role in limiting Th17 cell expansion and resulting EAE severity[38].

In summary, we report here that CD73 is expressed on a high proportion of Th17 cells during EAE development, including on cells in the CNS. However, CD73 deficiency did not affect differentiation, recruitment or function of Th17 cells as assessed by EAE clinical signs, flow cytometry and antigen recall assays. These data were unexpected given the known role of CD73 in regulating inflammatory immune responses, and suggest that in the face of a strong inflammatory stimulus, such as occurs during induction of EAE, the immunosuppressive role of CD73 becomes insufficient to prevent Th17 generation and onset of autoimmune inflammation.

## Materials and methods

### Mice

CD73^-/-^ and C57BL/6 (WT) mice were purchased from Jackson Laboratories and bred and housed under SPF conditions in an AAALAC-approved facility. All animal procedures were approved by the IACUC committee at the University of Pittsburgh. Mice were age and gender-matched within experiments, both male and female mice were used in all experiments, mice were used at 7–18 weeks of age.

### *In vitro* CD4^+^ T cell differentiation

CD4^+^ T cells from spleens and lymph nodes of naïve mice were purified by magnetic separation (Miltenyi Biotec, Germany). T cells were activated with 5 μg/ml plate-bound αCD3 (clone 145-TC11, BioXcell) in RPMI medium supplemented with 10% fetal bovine serum, 2 mM L-glutamine, 100 U/ml penicillin, 100 μg/ml streptomycin, and 50 μM 2-β-mercaptoethanol, HEPES and Na pyruvate. For Th17 differentiation, cells were cultured in the presence of recombinant mouse IL-1β (40 ng/ml), IL-23 (20 ng/ml), IL-6 (100 ng/ml), TGFβ1 (10 ng/ml); all cytokines from R&D Systems, MN. In all Th0 cell cultures 10 μg/ml αIFN-γ neutralizing antibodies (BioXcell) were added. For Th1 cultures, IL-12 (PeproTech, NJ) was added at 10 μg/ml. For Treg differentiation, T cells were cultured in the presence of recombinant mouse TGFβ1 (20 ng/ml), recombinant human IL-2 (100 U/ml) and αIFN-γ neutralizing antibodies (10 μg/ml).

### EAE induction

Naïve WT and CD73^-/-^ mice were immunized subcutaneously with 100μg MOG(35–55) (Bio synthesis, Lewisville, Texas, USA) emulsified in 200μl CFA (Difco Laboratories, Detroit, Michigan, USA) containing 100μg Heat Killed *Mycobacterium tuberculosis* H37Ra (Difco Laboratories, Detroit, Michigan, USA) distributed in four sites on the flanks. 200ng Pertussis toxin (List Biological Laboratories) was given intraperitoneally on day 0 and 2. Clinical scoring: Mice were monitored daily, and EAE clinical signs were scored according to the following grades: 1: flaccid tail; 2: impaired righting reflex and hindlimb weakness; 3: partial hindlimb paralysis; 4: complete hindlimb paralysis; 5: hindlimb paralysis with partial forelimb paralysis; 6: moribund/dead, in the reported experiments less than 4% of mice died from EAE. Cages in which mice were found to show signs of paralysis (grade 3 or higher) were provided access to food and water on the cage floor. At the end of the experiment, animals were euthanized by CO_2_ asphyxiation.

### Flow cytometry

The following FACS antibodies were purchased from BD Biosciences: CD4 (RM4-5), CD44 (IM7), Ki67 (B56), IFNγ (XMG1.2) and IL-17 (TC11-18H10). The following were purchased from eBioscience: CD73 (eBIOTY/11.8), RORγt (AKFJS9), Foxp3 (FJK-16s) and GM-CSF (MP1-22E9). For cytokine analysis, cells were cultured in complete medium (as described for T cell cultures above) with 50 ng/ml PMA and 500 ng/ml ionomycin (Sigma-Aldrich) in the presence of Golgiplug (BD Biosciences) for 3 to 4 hours followed by FACS staining and analysis. For intracellular cytokines, staining was performed using Cytofix-cytoperm kit from BD; RORγt and Foxp3 intracellular stains were performed using eBioscience Foxp3 staining kit according to manufacturer’s instructions. Prior to surface staining, cells were incubated for 20 min on ice with Ghost Dye^™^ Violet 510 (TONBO biosciences, CA) to allow exclusion of dead cells from analysis performed in FlowJo.

### MOG re-challenge and IL-17 ELISA

Draining lymph nodes from mice immunized with MOG(35–55) were processed to obtain single cell suspensions. Cells were cultured in flat bottom 96-well plates at a cell density of 1M cells/well with soluble αCD3 antibodies (clone 145-TC11, 5 μg/ml; BioXcell) in the presence or absence of IL-23 (20 ng/ml). IL-17 production was analyzed from culture supernatants three days after MOG-rechallenge using Ready-Set-Go ELISA kits (eBioscience). Samples were diluted accordingly using the diluent buffer included in the kits.

### Statistics

Parametric values were analyzed using Student’s *t*-test, or ONE-WAY ANOVA (with Tukey’s correction for multiple comparisons) when more than two groups were compared. EAE clinical scores and date of onset were analyzed using Mann-Whitney test (daily scores were analyzed separately). *P* values are shown as * = (p <0.05), ** = (p <0.01), *** = (p <0.001) and **** = (p <0.0001), where statistical significance was found.

## Supporting information

S1 File ARRIVE checklist. (PDF)

S2 File Excel of data used to generate figures. (XLSX)
